# Epilepsy and genetic in Rett syndrome: A review

**DOI:** 10.1002/brb3.1250

**Published:** 2019-03-30

**Authors:** Francesca Felicia Operto, Roberta Mazza, Grazia Maria Giovanna Pastorino, Alberto Verrotti, Giangennaro Coppola

**Affiliations:** ^1^ Child Neuropsychiatry Unit, Department of Basic Medical Sciences, Neuroscience and Sense Organs University of Bari “Aldo Moro” Bari Italy; ^2^ Child and Adolescent Neuropsychiatry, Medical School University of Salerno Fisciano Italy; ^3^ Department of Pediatrics University of L'Aquila Coppito Italy

**Keywords:** epilepsy, genetic, Rett syndrome

## Abstract

**Introduction:**

Rett syndrome (RTT) is a severe X‐linked neurodevelopmental disorder that primarily affects girls, with an incidence of 1:10,000–20,000. The diagnosis is based on clinical features: an initial period of apparently normal development (ages 6–12 months) followed by a rapid decline with regression of acquired motor skills, loss of spoken language and purposeful hand use, onset of hand stereotypes, abnormal gait, and growth failure. The course of the disease, in its classical form, is characterized by four stages. Three different atypical variants of the disease have been defined. Epilepsy has been reported in 60%–80% of patients with RTT; it differs among the various phenotypes and genotypes and its severity is an important contributor to the clinical severity of the disease.

**Methods:**

In this manuscript we reviewed literature on RTT, focusing on the different genetic entities, the correlation genotype–phenotype, and the peculiar epileptic phenotype associated to each of them.

**Results:**

Mutations in MECP2 gene, located on Xq28, account for 95% of typical RTT cases and 73.2% of atypical RTT. CDKL5 and FOXG1 are other genes identified as causative genes in atypical forms of RTT. In the last few years, a lot of new genes have been identified as causative genes for RTT phenotype.

**Conclusions:**

Recognizing clinical and EEG patterns in different RTT variants may be useful in diagnosis and management of these patients.

## 1 INTRODUCTION

Rett syndrome (RTT) is a severe X‐linked neurodevelopmental disorder first described in the medical literature more than 50 years ago: in 1966 Dr. Andreas Rett described 22 girls with a progressive neurological syndrome with seizures (Rett, 1966). Later, in 1983, Hagberg et al. imported the eponym Rett syndrome to 35 girls with similar characteristics, along with the first diagnostic criteria (Hagberg, Aicardi, Dias, & Ramos, 1983). In 1999, Amir et al. identified the genetic cause of RTT as a loss of function mutation in the gene Methyl‐CpG‐binding protein 2 (MECP2) (Amir et al., 1999). In 1994, Hagberg and Skjeldal developed more precise diagnostic criteria (Hagberg & Skjeldal, 1994); since then, clinical spectrum has been better clarified and diagnostic criteria have been modified in the last version of 2010 (Neul et al., 2010).

Rett syndrome primarily affects girls, with an incidence of 1:10,000–20,000 (Hagberg, Hanefeld, Percy, & Skjeldal, 2002). The diagnosis of RTT is based on clinical features, although, in clinical practice, gene analysis is performed to confirm it and to determine the causative mutation. Most children with RTT are the born from a normal pregnancy and delivery (Dolce, Ben‐Zeev, Naidu, & Kossoff, 2013). An initial period of apparently normal development (ages 6–12 months) is followed by a period of rapid decline with regression of acquired motor skills, loss of spoken language and purposeful hand use, onset of hand stereotypes, abnormal gait, and growth failure. This regression is sometimes sudden and often rapid, occurring in the time span of weeks to months and usually this period is associated with severe sleep disturbances, irritability, and poor eye contact (Dolce et al., 2013). According to revised diagnostic criteria for RTT by Neul et al., in classic RTT, a period of regression followed by recovery or stabilization and the presence of four main criteria and two exclusion criteria are required for diagnosis (Neul et al., 2010; Table 1). With regard to postnatal deceleration in head growth, it is an early sign, which begins between 2 and 4 months of age (Dolce et al., 2013). Nevertheless, it is not found in all patients with typical RTT (Hagberg, Stenbom, & Witt Engerstrom, 2000); for this reason, it has been eliminated from the necessary criteria, but it is considered nowadays a preamble to them, as a feature that should raise suspicion for the diagnosis (Neul et al., 2010).

**Table 1:** Revised clinical criteria for Typical RTT (Neul et al., 2010)

Main criteria	1. Partial or complete loss of acquired purposeful hand skills
	2. Partial or complete loss of acquired spoken language
	3. Gait abnormalities: impaired (dyspraxic) or absence of ability
	4. Stereotypic hand movements (hand wringing/squeezing, clapping/tapping, mouthing and washing/rubbing automatisms)
Exclusion criteria	1. Brain injury secondary to trauma (peri‐ or postnatally), neurometabolic disease, severe infections that causes neurological problems
	2. Grossly abnormal psychomotor development in first 6 months of life

The course of the disease, in its classical form, is characterized by four stages with an apparently normal early psychomotor development in the first 6 months of life (Hagberg et al., 2002; Table 2). In some cases, vague symptoms may be present, such as hypotonia, jerkiness, and reduced social interaction. Einspieler et al. carefully assessed videos of 22 Rett cases, examining movements, posture, and behavior during the first 6 months of life. Authors demonstrated an abnormal quality of general movements (100%), tongue protrusion (62%), postural stiffness (58%), asymmetric eye opening and closing (56%), abnormal finger movements (52%), hand stereotypies (42%), bursts of abnormal facial expressions (42%), bizarre smile (32%), tremor (28%), and stereotyped body movements (15%) (Einspieler, Kerr, & Prechtl, 2005).

**Table 2:** Clinical stages of typical RTT

Stage	Age	Clinical features
I. EARLY ONSET	6 months−1 year	hypotonia, delay in gross motor skills, loss of hand skills/speech, less eye contact, social interaction, and interest in toys
II. RAPID DESTRUCTIVE	1 year−3 years	autistic features, intellectual disability, hand stereotypes, motor dysfunction, respiratory abnormalities; microcephaly
III. PLATEAU	2 years−10 years	Seizures, improvement of behavior, eye contact, and hand use
IV. LATE MOTOR DETERIORATION	>10 years	further motor deterioration, spasticity/dystonia, scoliosis
IVa		Loss of the ability to walk
IVb		Never ambulant

In addition to the classical form, it has been recognized that some individuals present with many of the clinical features of RTT but do not have all the features of this condition. These have been termed “variant” or “atypical” RTT: the preserved speech variant (Zappella variant), the congenital variant (Rolando variant), and the early seizure variant (Hanefeld variant). For the diagnosis of atypical or variant RTT, a period of regression followed by recovery or stabilization and at least two of the four main criteria and five of the 11 supportive criteria are needed (Neul et al., 2010; Table 3). According to the data of the United States RTT National History Study, on a sample of 819 patients enrolled, 85.4% met diagnostic criteria for classic RTT and 14.6% for RTT variants (Percy et al., 2010).

**Table 3:** Diagnostic criteria for atypical or variant RTT (Neul et al., 2010)

Main criteria (2 out of 4)	1. Partial or complete loss of acquired purposeful hand skills
	2. Partial or complete loss of acquired spoken language
	3. Gait abnormalities: impaired (dyspraxic) or absence of ability
	4. Stereotypic hand movements (hand wringing/squeezing, clapping/tapping, mouthing and washing/rubbing automatisms)
Supportive criteria (5 out of 11)	1. Breathing disturbances when awake
	2. Bruxism when awake
	3. Impaired sleep pattern
	4. Abnormal muscle tone
	5. Peripheral vasomotor disturbances
	6. Scoliosis/kyphosis
	7. Growth retardation
	8. Small cold hand and feet
	9. Inappropriate laughing/screaming spells
	10. Diminished response to pain
	11. Intense eye communication‐“eye pointing”

Zappella variant is a clinical variant characterized by regression at 1–3 years, prolonged plateau phase, milder compromission of purposeful hand use, and milder intellectual disability. The distinctive feature of this variant is the recovery of language after regression, at a mean age of 5 years, along with the rarity of epilepsy. MeCP2 gene mutations are found in majority.

Rolando variant (or congenital variant) is characterized by severe psychomotor delay with inability to walk, severe postnatal microcephaly, regression in first 5 months, lack of typical RTT eye gaze, autonomic abnormalities (small cold hands and feet, peripheral vasomotor disturbances, breathing abnormalities while awake), and movement abnormalities (tongue stereotypes, jerky movements of the limbs). Mutations in MECP2 gene are rarely found in this variant, which is closely linked to mutations in FOXG1 (Forkhead box protein G1) gene.

Hanefeld variant is characterized by early onset of seizures (infantile spasms and refractory myoclonic epilepsy) before regression, usually before 5 months of life. Other typical RTT features are less frequent. Also, in this variant, mutations in the MECP2 gene are rarely found, as it is characterized by mutations in the CDKL5 (cyclin‐dependent kinase‐like 5) gene, a regulator of MECP2 which also has important roles in neuronal maturation and brain development.

RTT is the major cause of mental retardation in females after Down syndrome (Krajnc, 2015), but it has also been documented in normal female carriers, in females with variant forms, and in some males with Klinefelter syndrome, fatal encephalopathy, familial X‐linked mental retardation, and males with features of RTT (Neul et al., 2010). As an X‐linked disorder, RTT was considered lethal in males; however, males with clinical features of RTT were reported even before the discovery of the causal gene. Wan et al. first described a male patient with a congenital neonatal encephalopathy and mutation in MECP2, who died at 1 year of age (Wan et al., 1999). Males with MECP2 mutations fall into four categories: severe neonatal encephalopathy and infantile death; classical RTT; less severe neuropsychiatric symptoms; MECP2 duplication syndrome (Imessaoudene et al., 2001; Jülich, Horn, Burfeind, Erler, & Auber, 2009; Kyle, Vashi, & Justice, 2018; Meloni et al., 2000; Ramocki et al., 2009; Schwartzman, Bernardino, Nishimura, Gomes, & Zatz, 2001; Topҫu et al., 2002; Van Esch et al., 2005; Zeev et al., 2002; Table 4).

**Table 4:** Clinical features and genetic profile in males with MECP2 mutations (Kyle et al., 2018)

	Genetic profile	Clinical features
1. Severe neonatal encephalopathy and infantile death	MECP2 mutations inherited from mildly symptomatic or asymptomatic mothers	If born, severe neonatal encephalopathy with respiratory arrest and seizures and death within 2 years of age
2. Classical RTT	XXY karyotype or other somatic mosaicisms	Like RTT clinical features in female patients
3. Less severe neuropsychiatric symptoms	MECP2 mutations less severe than those in female patients	Intellectual disability and motor abnormalities (broad spectrum of symptoms and possible overlap with features of Angelman syndrome)
4. MECP2 duplication syndrome	Gain of MECP2 dosage	Hypotonia, severe intellectual disability, lung infections, seizures, absent or limited speech and walking, motor spasticity, and muscle stiffness

## 2 CURRENT UPDATE IN THE GENETIC OF RETT SYNDROME

It is well established that mutations in MECP2 gene, located on Xq28, account for 95% of typical RTT cases and 73.2% of atypical RTT (Ehrhart, Sangani, & Curfs, 2018). MECP2 is a protein ubiquitously present throughout all human tissues and particularly abundant in neurons and astrocytes in the brain. It is expressed at low levels prenatally and increases during neuronal maturation and synaptogenesis suggesting an important role in neuronal activity and plasticity (Cohen et al., 2003; Jung et al., 2003; Samaco, Nagarajan, Braunschweig, & LaSalle, 2004). The exact mechanism by which loss of MECP2 results in the distinctive clinical features of RTT remains uncertain; it is supposed that abnormal cortical glutamatergic synaptic responses and excitatory connectivity result in excess of inhibition and deficits in neuronal plasticity (2012). MECP2 mutations usually occur de novo: in >99% of cases these mutations occur sporadically, in less than 1% are inherited from one parent (Dolce et al., 2013); familial cases originate from mutations inherited from healthy or mildly affected mothers or from gonadal mosaicism (Matijevic, Knezevic, Slavica, & Pavelic, 2009). Majority of patients is therefore heterozygous for MECP2 mutation, carrying one normal and one mutated copy of the gene (Kyle et al., 2018; Neul et al., 2008). Most of the de novo mutations occur almost exclusively in the paternal gamete (Trappe et al., 2001). Currently, about 800 different mutations of MECP2 gene causing RTT have been identified: point mutations, insertions, duplications, small or large deletions in almost all parts of the gene (Ehrhart et al., 2018). Majority of causative mutations has been found in eight single‐nucleotide polymorphism hotspots as missense and nonsense mutations: T158M; R255X; R168X; R306C; R294X; R270X; R133C (Kyle et al., 2018; Percy et al., 2010). These mutations account for approximately 70% of all mutations (Kyle et al., 2018) and R168X is the most common one; C‐terminal deletions account for 8% and large deletions for another 5% (Neul et al., 2008).

Despite the wide spectrum of MECP2 mutations and the variety of phenotype severity, there are no clear genotype–phenotype correlations (Guerrini et al., 2012). Some studies in literature have proposed a correlation between genotype and clinical features in RTT patients (Cuddapah et al., 2014; Neul et al., 2008). Early truncating mutations such as R168X, R255X, and R270X and large insertions and deletions cause the most severe phenotype. Missense mutations such as R133C and R306C, late truncating mutations such as those in R294X and others in the 3’ end are associated with the mildest phenotype. Despite this, phenotype variations commonly occur between patients with the same mutation. Some possible explanation has been proposed, as differences in X chromosome inactivation (Ehrhart et al., 2018; Knudsen et al., 2006) and the presence of a second gene mutation, acting as modifier, alleviating or enhancing the phenotype outcome (Zeev et al., 2002).

RTT was diagnosed also in 3%–5% patients who were negative for MECP2 mutations (Neul et al., 2008). CDKL5 and FOXG1 are other genes identified as causative genes in atypical forms of RTT, respectively the Hanefeld Variant and Rolando variant (Ariani et al., 2008; Weaving et al., 2004).

CDKL5 is a gene located on Xp22 and code for a ubiquitous protein, mainly expressed in the brain, thymus, and testes, with a role in neuronal maturation (Guerrini et al., 2012; Rusconi et al., 2008). Several mutations in the CDKL5 gene have been identified: point mutations, sequence variations resulting in missense, nonsense, splice and frameshift mutations, microdeletions, and larger rearrangements (Guerrini et al., 2012). CDKL5 is involved in the early seizure variant of RTT (Mari et al., 2005; Scala et al., 2005).

FOXG1 is a transcriptional repressor involved in neuronal differentiation and highly expressed in the developing brain, whose gene is located on chromosome 14q12. Mutations in FOXG1 are responsible for the congenital variant of RTT (Ariani et al., 2008). Patients with FOXG1 mutations (duplications/deletions, nonsense, frameshift and missense mutations) have clinical features characterized by postnatal growth deficiency and microcephaly, developmental delay with absent speech, defective social reciprocity, poor sleep, stereotypes, dyskinesia, and severe early onset epilepsy (Brunetti‐Pierri et al., 2011; Guerrini et al., 2012; Yeung et al., 2009).

In the last few years, using whole exome sequencing, many other uncommon genetic mutations in 69 new genes have been identified as causative for RTT phenotype (classic or variant) (Ehrhart et al., 2018; Table 5).

**Table 5:** New causative genes identified in the last few years as causative genes for RTT phenotype (review of recent literature)

Gene	Reference
SMC1A	Glissen et al. (2014), Huisman et al. (2017)
TBL1XR1	Saitsu et al. (2014)
GABRD	Okamoto et al. (2015)
SCN2A	Baasch et al. (2014)
SHANK3	Hara et al. (2015)
SCN8A, IQSEC2	Olson et al. (2015)
WDR45	Hoffjan et al. (2016)
JMJD1C	Saez et al. (2016)
SATB2	Lee et al. (2016)
GABBR2	Yoo et al. (2017)
IQSEC2, KCNA2	Allou et al. (2017)
ANKRD31, CHRNA5, HCN1, SCN1A, TCF4, GRIN2B, SLC6A1, MGRN1, BTBD9, SEMA6B, AGAP6, MGRN1, VASH2, ZNF620, GRAMD1A, GABBR2, ATP8B1, HAP1, PDLIM7, SRRM3, CACNA1I	Lucariello et al. (2016)
TCF4, EEF1A2, STXBP1, ZNF238, SLC35A2, ZFX, SHROOM4, EIF2B2, RHOBTB2, SMARCA1, GABBR2, EIF4G1, HTT	Lopes et al. (2016)
PWP2, SCG2, IZUMO4, XAB2, ZSCAN12, IQSEC2, FAM151A, SYNE2, SMC1A, ARHGEF10L, HDAC1, TAF1B, KCNJ10, CHD4, LRRC40, LAMB2, GRIN2B, IMPDH2, SAFB2, ACTL6B, STXBP1, TRRAP, WDR45, SLC39A13, FAT13, IQGAP3, NCOR2, GABRB2, TCF4, GRIN2A	Sajan et al. (2017)
GRIN2B, GABBR2, MEF2C, STXBP1, KCNQ2, SLC2A1, TCF4, SCN2A, SYNGAP1, CACNA1I, CHRNA5, HCN1	Vidal et al. (2017)
STXBP1	Yuge et al. (2018)
CTNNB1, WDR45	Percy et al. (2018)
SCNA1	Henriksen, Ravn, Paus, Tetzchner, and Skjeldal (2018)

In classical or atypical clinical variants of RTT, a PCR‐mutation screening of exons 3 and 4 of MECP2 gene should be first performed and, in the case of negativity, further screening for large deletions or small MECP2 mutations in exons 1 and 2 should be carried on.

In case of suspected early onset Rett syndrome, with epileptic seizures or spasms or microcephaly even in males, CDKL5 mutation screening and FOXG1 should follow.

In case of negativity, targeted resequencing consisting of ad hoc gene panels or whole exome sequency should then be recommended, together with an array CGH analysis to exclude microdeletions/microduplications on the whole chromosomal set. The high banding karyotype may have priority in males with Rett‐like phenotype and possible XXY aneuploidy.

## 3 EPILEPSY IN RETT SYNDROME

Epilepsy has been reported in 60%–80% of patients with RTT, differing among the various phenotypes and genotypes. Incidence is higher in patients with early onset RTT and more severe developmental disabilities (greater impairment of ambulation, hand use, and communication) (Glaze et al., 2010). On the other hand, the severity of epilepsy is an important contributor to the clinical severity of RTT phenotype (Krajnc, 2015). Seizures seem to occur earlier in those patients who do not have MECP2 mutations (Jian et al., 2006).

There is not a characteristic “first seizure” semiology in Rett syndrome (Dolce et al., 2013) and all seizure types have been reported (Krajnc, 2015). Complex partial and generalized tonic‐clonic are the commonest seizure types in RTT (Pintaudi et al., 2010), whereas absence and clonic seizures are less frequent (Buoni et al., 2008; Steffenburg, Hagberg, & Hagberg, 2001). Moreover, also early febrile seizures seem to be more frequent in RTT compared with general population (12% vs. 2%–5%) (Nissenkorn et al., 2010).

Steffenburg et al. have suggested that early onset of seizures can be associated with more seizure types, intractable epilepsy, and status epilepticus (Steffenburg et al., 2001), whereas Nissenkorn et al. stated that seizure onset after 5 years is a good prognostic factor (Nissenkorn et al., 2010). Reviewing literature, beyond the age of onset, other factors have been identified as risk factors for epilepsy and its severity: microcephaly (Steffenburg et al., 2001), developmental delay in the first 10 months of life, and absence of walking (Jian et al., 2006), certain mutations in the MECP2 gene with regard to classical form (Glaze et al., 2010).

Despite its frequent presence in Rett syndrome, epilepsy can be difficult to diagnose as many other clinical manifestations could be mistaken as seizures. According to Guerrini et al., the overall occurrence of epilepsy in Rett syndrome is probably overestimated and in their sample of 389 patients, only 48% could be considered as having true epileptic seizures (Guerrini et al., 2012). Glaze et found that, among parent‐reported seizure behaviors, only one third was temporally associated with epileptiform abnormalities on electroencephalogram (EEG) (Glaze et al., 2010). In RTT patients, several behaviors could be wrongly classified as epileptic seizures: hand stereotypies, breath‐holding and cyanosis, hyperventilation and chaotic breathing pattern in the waking state, staring, oculogyric movements, blinking episodes, oral facial dyskinesias, bouts of laughing or screaming, tremor, dystonia, jerking, spasticity, and episodic atonia. Obviously, a misdiagnosis can lead to patient's overtreatment and even pseudo drug‐resistance.

EEG is an important diagnostic tool because it allows to distinguish true seizures from nonepileptic events. However, it is important to consider that EEG can be abnormal in Rett syndrome, even without the presence of epilepsy in comorbidity; in such cases a prolonged video EEG could help clinicians (Dolce et al., 2013).

According to Tarquinio et al., because EEG is almost universally abnormal after age 3 in Rett syndrome, clinicians should not prescribe antiseizure medications for patients without clear epileptic seizures, despite an abnormal EEG (Tarquinio et al., 2017).

### 3.1 Epilepsy on MECP2‐positive patients

With regard to classical form, seizures usually appear in the II or III stage of the disease, with the highest frequency occurring between 7 and 12 years (Krajnc, 2015).

Reviewing literature, severe mutations in MECP2 gene, such as large deletions, early truncating, and missense mutations in the methyl‐binding domain or the nuclear localizing segment seem to be associated with earlier or more sever epilepsy, whereas milder mutations such as late truncating or C‐terminal deletions seem to have a protective effect on epilepsy onset (Jian et al., 2006, 2007; Nectoux et al., 2008; Pintaudi et al., 2010).

Jian et al., in 2006, investigated risk factors for seizure onset in Rett syndrome in a sample of 288 patients. They found that R168X, R294X, and C‐terminal mutations in MECP2 gene, conferred a protective factor for seizure onset before 4 years of age, whereas X inactivation seemed to be a protective factor against seizure onset before 48 months of life. Authors also found that, among MECP2 mutations, the median age of seizure onset varied from 37 months in those patients with R255X mutation to 76 months in those with R294X (Jian et al., 2006).

Pintaudi et al. found that epilepsy was more frequent in patients with large MECP2 deletions and R294X mutation and it occurred less frequently in subjects with no identified mutations and C‐terminal deletions in MECP2. The latter proved to be a protective factor, with respect to both the occurrence of epilepsy and drug resistance. Moreover, patients with large MECP2 deletions started having seizure later (6.72 years), rather than those patients with other types of mutations, particularly R168X and R255X (Pintaudi et al., 2010).

In 2012, Guerrini et al. examined 389 patients with RTT, including 315 classical and 74 atypical Rett patients. Authors found that occurrence of seizures was comparable in the two groups (60% vs. 61%), with MECP2 identified in 90% of patients (93% of the classical Rett subgroup) and seizures reported in 59% of those patients with MECP2 mutations and in 73% of those without MECP2 mutations. In the first subgroup, 60% of patients had a mutation in one of the eight most common mutations (R106W, R133C, T158M, R168X, R255X, R270X, R294X, R160C), 9% had C‐terminal deletions and 8,5% had large deletions. Among all, seizures were more frequent in patients with T158M mutation (Guerrini et al., 2012). This finding is consistent with a previous one by Glaze et al., who found significantly more epilepsy in patients with T158 mutation and R106W, than subjects with R255X or R306C mutations (Glaze et al., 2010). Furthermore, Buoni et al. had formerly reported a higher prevalence of the T158 mutation in drug‐resistant epilepsy (Buoni et al., 2008).

### 3.2 Epilepsy on CDKL5‐positive patients

The early seizure variant (Hanefeld variant), associated with CDKL5 mutations, is characterized by seizure onset before 5 months of age, usually with infantile spasms (50%) and refractory myoclonic epilepsy (25%) (Dolce et al., 2013; Pintaudi et al., 2010).

In RTT associated with CDKL5 mutations, epilepsy is typically manifested as an epileptic encephalopathy, with infantile spasm, multifocal and myoclonic seizures. Bahi‐Buisson et al. have described a three‐step epilepsy phenotype associated with CDKL5 mutations in 13 girls aged from 2.5 to 19 years: early epilepsy (stage I), then epileptic encephalopathy (stage II) and finally, late multifocal and myoclonic epilepsy (stage III). Moreover, according to the Authors, patients with mutations involving the catalytic domain of the protein show earlier onset and intractable infantile spasms and more severe late onset multifocal and myoclonic epilepsy than patients with truncating mutations downstream of the catalytic domain (Bahi‐Buisson et al., 2008).

### 3.3 Epilepsy on FOXG1‐positive patients

Severe early onset epilepsy has been reported as main features of the FOXG1‐related phenotype (Guerrini et al., 2012). Kortüm et al. have described 11 patients carrying mutations in FOXG1 gene documenting tonic, generalized tonic‐clonic and partial seizures, with onset between 3 months and 6 years (Kortüm et al., 2011). Moreover, the association between 14q12 duplication including FOXG1 and infantile spasms has been documented (Striano et al., 2011), suggesting that overexpression of FOXG1 could have a specific role in the pathogenesis of infantile spasms.

## 4 EEG FINDINGS

**Figure 1:**
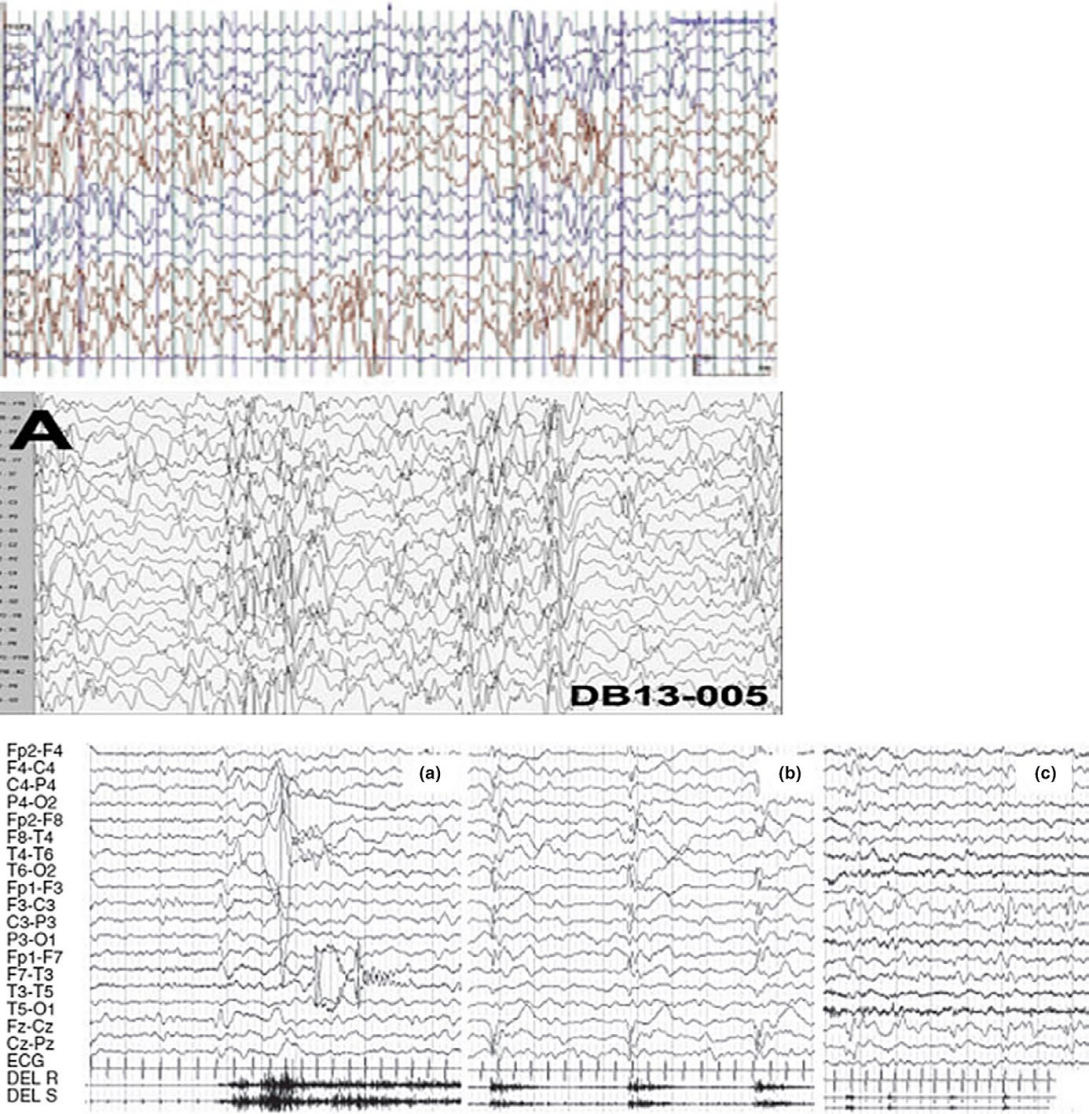
EEG pattern of (a) a classical Rett syndrome, (b) a child with FOXG1 microduplication and epileptic spasms and microcephaly, (c) a 4‐month CDKL5 girl with tonic/vibratory seizure followed by a cluster of spasms and clonic jerks

In literature, there are few reports of the characteristics EEG findings in patients with Rett syndrome, especially with regard to atypical variants (Figure 1).

### 4.1 EEG on MECP2‐positive patients

In classical form, EEG pattern progress through the four clinical stages of the disease, however with abnormalities that are not diagnostic for Rett syndrome. Very early in the disease, in Stage 1, seizures are not a prominent feature and EEGs typically tend to be normal. In Stage 2, focal spikes in the centrotemporal regions may be seen, with a characteristic involvement of the motor cortex that correlates with the clinical onset of motor abnormalities; progressively, sleep abnormalities appear. Seizure burden is prominent in Stage 3; in this stage, EEG is characterized by abnormal sleep patterns, bilaterally synchronous bursts of pseudoperiodic delta activity, and generalized rhythmic spike discharges. In stage 4, seizures are no longer a prominent feature; EEGs show slowing of background activity, theta activity in central and frontal regions, multifocal epileptiform activity in awake state, and generalized slow spike‐wave activity in sleep (Dolce et al., 2013; Moser, Weber, & Lutschg, 2007).

EEG findings that have been described as correlate of these ictal events are bilateral and synchronous initial flattening, followed by repetitive sharp waves and spikes; generalized ictal patterns in neonates or very young infants, atypical hypsarrhythmia, slowing background with multifocal interictal discharges, and suppression burst pattern (Grosso et al., 2007; Guerrini et al., 2012; Melani et al., 2011).

### 4.2 EEG on CDKL5‐positive patients

Limited information is available regarding the precise electroclinical phenotype in epileptic patients with mutations in CDKL5 gene. Bahi‐Buisson et al. have reported the electroclinical description of epilepsy in 12 patients with pathogenetic CDKL5 mutations. Authors found that early EEG showed generalized flattening of the tracing with a fast activity discharge followed by spikes and waves in both frontal and central regions in stage I; stage II EEG was characterized by modified hypsarrhythmia or very slow activity consisting of bilateral high‐amplitude delta waves, intermixed with spikes and polyspikes on both frontal, central, or occipital regions; during stage III, myoclonic seizures were associated with isolated polyspikes and wave complex or with fast activity on both central regions, whereas interictal EEG showed high‐amplitude spikes, polyspikes, and waves in central, temporal, or temporooccipital regions (Bahi‐Buisson et al., 2008).

### 4.3 EEG on FOXG1‐positive patients

To our knowledge, there are few reports concerning EEG findings in patients with RTT phenotype associated with FOXG1 mutations. Available EEG data only report focal and multifocal abnormalities in patients with tonic, generalized tonic‐clonic and complex partial seizures (Guerrini et al., 2012; Kortüm et al., 2011).

## 5 CONCLUSIONS

Rett syndrome is a truly complex neurological disorder, whose diagnostic criteria are based on phenotypic description. In addition to the classical form, a heterogeneous spectrum of phenotypes identifies those forms called “atypical Rett”. Nowadays, it seems more correct to consider this broad spectrum of neurological disorders as the expression of a complex encephalopathy. Many efforts have been made to understand the molecular bases that underlie all different clinical phenotypes of RTT. With the increase of knowledge, especially thanks to recent genetic advances, many new genes have been identified as causative for RTT phenotype, in addition to MECP2, CDKL5, and FOXG1.

Epilepsy is a prominent symptom in RTT and it substantially contributes to the severity of the disease. Some genotypes are reported to be more frequently associated with epilepsy and even with a drug‐resistant course. Recognizing clinical and EEG patterns in different RTT variants may be useful in diagnosis and management of these patients. Long‐term video EEG is surely a useful diagnostic tool which allows to differentiate between nonepileptic paroxysmal events and real epileptic seizures, to further classify seizure semiology and finally choice the most appropriate drug treatment. On the other hand, the identification of individual genetic background allows to diagnose the disorder properly and is helpful for treatment, not only for the genetic counseling.

In this manuscript, we aimed to clear‐up the long‐standing misunderstanding of differential diagnosis between Rett syndrome associated with MECP2 mutations and Rett‐like phenotype of other neurodevelopmental diseases associated with different emerging genes.

Comparative studies with large cohorts of patients are needed to better understand the relationship between genotype and phenotype and correctly diagnose and treat these patients.

## CONFLICT OF INTEREST

None.
